# ﻿An updated checklist of Mozambique’s vascular plants

**DOI:** 10.3897/phytokeys.189.75321

**Published:** 2022-01-28

**Authors:** Délcio Odorico, Enrico Nicosia, Castigo Datizua, Clayton Langa, Raquel Raiva, Joelma Souane, Sofia Nhalungo, Aurélio Banze, Belkiss Caetano, Vânia Nhauando, Hélio Ragú, Mário Machunguene Jr, Jónata Caminho, Leonel Mutemba, Efigénio Matusse, Jo Osborne, Bart Wursten, John Burrows, Silvio Cianciullo, Luca Malatesta, Fabio Attorre

**Affiliations:** 1 Department of Biological Sciences, Eduardo Mondlane University, Av. Julius Nyerere 3534, P.O. Box 257, Maputo, Mozambique Eduardo Mondlane University Maputo Mozambique; 2 Department of Environmental Biology, Sapienza – University of Rome, Piazzale Aldo Moro 5, 00185, Roma, Italia University of Rome Roma Italy; 3 Mozambique Agricultural Research Institute, Av. FPLM 2698, P.O. Box 3658, Mavalane, Maputo, Mozambique Mozambique Agricultural Research Institute Maputo Mozambique; 4 Wildlife Conservation Society, Rua Faustino Vanombe 61, P.O. Box 421, Maputo, Mozambique Wildlife Conservation Society Maputo Mozambique; 5 Royal Botanic Gardens, Kew, Richmond, Surrey, TW9 3AB, UK Royal Botanic Gardens Richmond United Kingdom; 6 Meise Botanic Garden Herbarium, Nieuwelaan 38, Meise 1860, Belgium Meise Botanic Garden Herbarium Meise Belgium; 7 Buffelskloof Nature Reserve and Herbarium, P.O. Box 710, Lyndenburg, Mpumalanga Province, South Africa Buffelskloof Nature Reserve and Herbarium Lyndenburg South Africa

**Keywords:** Biodiversity, checklist, flora, Mozambique, taxonomy, vascular plants

## Abstract

An updated checklist of Mozambique’s vascular plants is presented. It was compiled referring to several information sources such as existing literature, relevant online databases and herbaria collections. The checklist includes 7,099 taxa (5,957 species, 605 subspecies, 537 varieties), belonging to 226 families and 1,746 genera. There are 6,804 angiosperms, 257 pteridophytes, and 38 gymnosperms. A total of 6,171 taxa are native to Mozambique, while 602 are introduced and the remaining 326 taxa were considered as uncertain status. The endemism level for Mozambique’s flora was assessed at 9.59%, including 278 strict-endemic taxa and 403 near-endemic. 58.2% of taxa are herbaceous, while shrubs and trees account respectively for 26.5% and 9.2% of the taxa. The checklist also includes ferns (3.6%), lianas (1.7%), subshrubs (0.5%) and cycads (0.3%). Fabaceae, Poaceae and Asteraceae are the three most represented families, with 891, 543 and 428 taxa, respectively. The extinction risk of 1,667 taxa is included, with 158 taxa listed as Vulnerable, 119 as Endangered and as 24 Critically Endangered. The geographical distribution, known vernacular names and plants traditional uses are also recorded.

## ﻿Introduction

Mozambique lies on the southeast coast of Africa, between latitudes 10°27'S to 26°52'S and longitudes 40°51'E to 30°12'E, bordered by Tanzania in the north, the Indian Ocean in the east, Zambia to the northwest, Malawi, Zimbabwe, and Eswatini to the west and South Africa to the west and south. The country has a total area of 801,590 km^2^ (Instituto Nacional de Estatística 2020), about 70% of which is covered by forests or other woody vegetation and 26% is included in conservation areas, such as national parks, reserves and game farms (Ministry for the Coordination of Environmental Affairs 2014).

The country is an important area of plant biodiversity (Conde et al. 2014), deriving its plant richness from geomorphological and climatic factors (Darbyshire et al. 2019). Geographically, the Zambezi River, crossing Mozambique from west to east towards the Indian Ocean, roughly bisects the country into two main regions: a southern region, dominated by lowlands, and a northern region which consists of a large plateau. The highland region in Manica Province encompasses the highest point in the country, rising up to 2,436 meters at Mount Binga (Ministry for the Coordination of Environmental Affairs 2014). The country’s underlying geology can also be broadly divided into two different regions: the southern region of Mozambique consist of sedimentary rocks (Rutten et al. 2008), whereas the ancient granite rock basement of Africa underlies most of northern and west-central regions (Boyd et al. 2010). The climate is tropical over most of the country, with a sub-tropical climate in the south. Mozambique has two main seasons: a cooler dry season, from April to October and a warmer humid season from October to April (Barbosa et al. 2001). The northern region (Niassa, Cabo Delgado, Nampula and Zambézia provinces) has higher temperatures, with annual temperature averages of 25.5 °C in the coastal area dropping to 18 °C in the uplands. Central Mozambique (Tete, Manica and Sofala provinces) exhibits mean annual temperatures of 25 °C for the coastal lowlands and 20°C for the interior highlands. In the south (Inhambane, Gaza and Maputo provinces) the average annual temperatures vary from 23 °C in the coastal area to 25 °C in the interior (Wils and Lutz 2002). Rainfall distributions fluctuate widely through the country, following a north-south gradient with higher rainfall in the north and in the mountainous areas, where average annual rainfall can be up to 2000 mm. The annual average precipitation is 1030 mm, ranging from 1400 mm/year near the Zambezi basing to 300 mm/year in the south (Uamusse et al. 2017).

**Figure 1.**
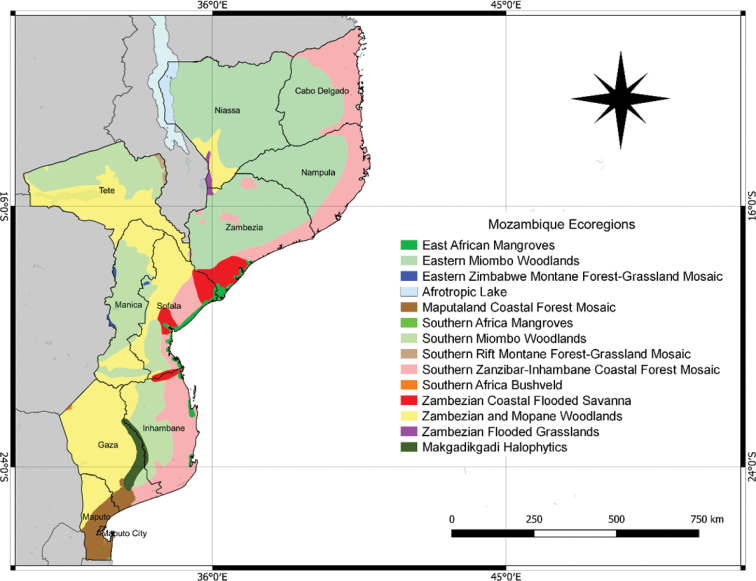
Mozambique ecoregions (Burgess et al. 2004; Olson 2020).

According to Burgess et al. (2004), thirteen ecoregions are recorded in Mozambique (Fig. 1). These are included in five main biomes: arid and semi-arid forest; tropical and subtropical rangelands, savannas, shrublands, and woodlands; flooded grasslands and savannas; mountain grasslands and shrublands; mangroves (Ministério da Terra Ambiente e Desenvolvimento Rural 2015). Such biogeographical complexity results in high plant diversity. Until now, only 6,264 plants species have been recorded (Hyde et al. 2021) and seven broad vegetation communities identified, namely: miombo, woodland, mopane woodland, undifferentiated woodland, afromontane communities, halophytic vegetation, swamp vegetation and coastal mosaic (Bandeira et al. 1996).

**Figure 2.**
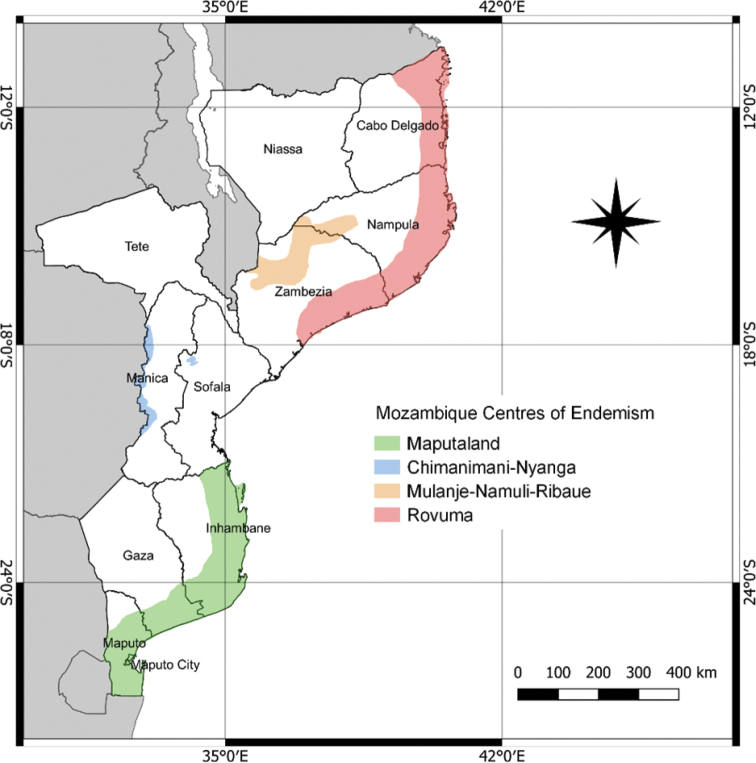
Mozambique Centres of Endemism. Modified version from Darbyshire et al. (2019).

According to Darbyshire et al. 2019, Mozambique has a relatively high level of plant endemism, with 572 taxa classified as strict-endemic or near-endemic, which are not evenly distributed throughout the country. Approximately 80% of Mozambique is included in the Zambezian Regional Centre of Plant Endemism (Bandeira et al. 1996), a continental scale phytochorion including the whole of Zambia, Malawi, Zimbabwe, large parts of Angola, Tanzania and Mozambique, and some small parts of Zaire, Namibia, Botswana, and South Africa (White 1983). Focusing on more restricted phytogeographical units, Darbyshire et al. (2019) has proposed four main Centres of Plant Endemism (Fig. 2). The Rovuma Centre of Endemism, previously referred as the Lindi Centre of Endemism (Clarke 1998) and restricted to southeast Tanzania, has recently been extended to the north Mozambique encompassing the coastal area of Cabo Delgado, Nampula and Zambézia provinces (Burrows and Timberlake 2011; Darbyshire et al. 2019). The Maputaland Centre of Endemism, shared with the KwaZulu-Natal province of South Africa and Eswatini, extends from the coastal lowlands of southern Mozambique to the Save River (Van Wyk 1996; Darbyshire et al. 2019). This centre can be subdivided into at least three sub-centres, such as the Maputaland (sensu stricto), the Lebombo Mountains Centre and the Inhambane Centre (Darbyshire et al. 2019). As part of the Afromontane Archipelago-like Centre of Endemism, Mozambique shares the Chimanimani-Nyanga Centre of Endemism with neighbouring Zimbabwe (Clark et al. 2017; Darbyshire et al. 2019), and includes large part of the Mulanje-Namuli-Ribáuè Centre of Endemism, which extends from southern Malawi to Zambézia and Nampula provinces (Darbyshire et al. 2021).

Despite its high diversity, the Mozambican flora has received only limited research coverage, remaining poorly known (Ministry for the Coordination of Environmental Affairs 2014; Darbyshire et al. 2019). Until the historical Botanic Mission to Mozambique (1942 – 1948) which represents the first study of the country’s plant diversity, the Mozambican flora was little known (Conde et al. 2014). The Botanic Mission to Mozambique represented a breakthrough for botanical studies in the country (Ministry for the Coordination of Environmental Affairs 2014), enabling the collection of more than 7,600 herbarium samples and the description of many families and species (Conde et al. 2014). The country’s protracted instability caused by the war of independence (1964 – 1975) and the subsequent civil war (1977 – 1992) resulted in a long period in which biodiversity research was neglected. However, in the last two decades a new impetus in botanical studies has risen (Cheek et al. 2018). In the early 2000s, da Silva et al. (2004) published a preliminary checklist of Mozambique’s vascular plants, which included 3,932 indigenous species. The checklist was built on the analysis of specimens from the National Herbarium of Mozambique (LMA) and the Eduardo Mondlane University (LMU) with additional records from literature sources. However, the list was under-representative of Mozambique’s plant diversity. Subsequent surveys provided new species and new country records. Timberlake et al. (2011) documented 68 new taxon records for Mozambique during a survey of the coastal dry forests in the Cabo Delgado Province in the northeast Mozambique, while Harris et al. (2011) listed another 31 new taxa for the country. The recent research and programme, such as the ongoing “Flora Zambesiaca” series, has progressively increased the estimated number of species in Mozambique and produced a huge effort to document the country’s floristic diversity. According to the Flora of Mozambique website (Hyde et al. 2021), currently the most comprehensive database for plant diversity in Mozambique, 6,264 species are recorded in the country. This figure is expected to grow rapidly, following the increase in botanical expeditions and the resulting new discoveries (Cheek et al. 2018; Darbyshire et al. 2019), marking Mozambique amongst the countries with the highest rate of discovery of new species in continental Africa (Darbyshire et al. 2020). On this basis, considering the crucial role of the national floristic inventory to assess plant conservation, management, and ecological restoration (Brundu and Camarda 2013; Lorite 2016), the need for an updated checklist to summarise the current state of knowledge of Mozambican vascular flora clearly emerges.

This paper presents an updated checklist of Mozambique’s vascular plants serving as a basis to guide further botanical research and to support biodiversity conservation planning. For each listed taxon, data such as the nomenclature, taxonomic classification, distribution, local occurrence details, life forms, endemism, and extinction risk are reported. Moreover, considering the key role of indigenous names and traditional uses of plants in botanical studies, frequently conducted through field surveys carried out with local people (de Koning 1993), the known vernacular names and traditional uses of the listed species are reported. Data were collected from large and freely available biodiversity databases, herbarium, museum collections (both national and foreign), and literature sources.

## ﻿Methods

### ﻿Data collection and organisation

To compile the present checklist, multiple information sources were examined and combined. An initial list (*n* = 3,932 species) was obtained, after verification of the taxa’s nomenclature, from da Silva et al.’s preliminary checklist (2004). The initial list was extended using data from the Buffelskloof Herbarium (BNRH, *n* = 4,266 records), the Royal Botanic Gardens Kew Herbarium (K, *n* = 7,484 records), the National Herbarium of Mozambique (LMA, *n* = 22,703 records) and the Eduardo Mondlane University’s Herbarium (LMU, *n* = 2,936 records) (acronyms according to Thiers 2020). Successively, we included taxa described in Mozambique from the following relevant databases of plant diversity and taxonomic research: Global Biodiversity Information Facility (GBIF, https://doi.org/10.15468/dl.gq7jnb, *n* = 91,832 records), Plants of World Online (POWO, http://www.plantsoftheworldonline.org/*n* = 5,639 species), Flora of Mozambique (https://www.mozambiqueflora.com/, *n* = 6,264 species), Flora Zambesiaca (http://apps.kew.org/efloras/advsearch.do?reset=true, *n* = 4,482 species), and JSTOR – Global Plants (https://plants.jstor.org/, *n* = 1,846 species). Finally, additional taxa were found through the review of previous studies on Mozambique’s flora (de Koning 1993; Timberlake et al. 2007, 2009; Wursten et al. 2017; Bayliss et al. 2010; Müller et al. 2012; Burrows et al. 2018; Darbyshire et al. 2019, *n* = 4,468 species) and through an extensive review of the most relevant ethnobotanical studies (Krog et al. 2006; Bandeira et al. 2007, 2011; Ribeiro et al. 2010; Silva et al. 2011; Williams et al. 2011; Bruschi et al. 2011, 2014; Conde et al. 2014; Santo-António and Goulão 2015; Moura et al. 2018; Barbosa et al. 2020; Manuel et al. 2020, *n* = 394) to document the traditional knowledge associated with the use of plants in the country.

Overall, a list of 157,576 records was produced, on which a thorough refinement procedure was performed through a Microsoft Excel 2010 spreadsheet. All records were organised by family rank, based on the classification system proposed by APG IV (Angiosperm Phylogeny Group 2016) for the angiosperms, by PPG I (Pteridophyte Phylogeny Group 2016) for the pteridophytes and by Christenhusz et al. (2011) for the gymnosperms. Taxa at rank of form and hybrids were not considered. Different quality filters were applied to remove repeated taxa and to exclude fungi, lichens, algae, bryophytes, and marine species. Finally, a manual refinement was carried out to clean repetition, remove doubtful taxa (labelled as “aff.”, “cf.” and “sp.”) and those whose taxonomic status was uncertain. The resulting intermediate list consisted of 15,605 taxonomic names, 9.9% of the initial collection.

### ﻿Taxonomic validation

The obtained list was subjected to a taxonomic validation process. Taxonomic rank and plant names were verified and validated with international reference databases: POWO (2021), African Plant Database (2021), The Plant List (2013). Version 1.1 and World Flora Online (2021). Subsequently, data review and validation was performed by floristic experts from the Royal Botanic Gardens Kew, the Botanic Garden Meise and the Buffelskloof Research Centre, which have verified the accepted species name derived from reference databases. Errors and inconsistencies found in the process (such as taxonomic misidentification, geographic errors, and incorrect life form) were assessed and corrected.

### ﻿Checklist outline

For each entry in the checklist, the taxonomic rank (species, subspecies, variety) is reported. A pragmatic approach was taken when treating the data records for infraspecific taxa (subspecies, varieties), autonyms and inclusive species names. To avoid artificially increasing the overall number of taxa in the checklist, inclusive species names were excluded where possible for species with infraspecific taxa occurring in Mozambique. However, in some cases, we retained inclusive species names where the infraspecific taxon was unclear and the data record added useful distribution information.

Using a modified classification system derived from Darbyshire et al. (2019), we categorised the plants listed in one of the following seven life forms categories: tree, shrub, subshrub, liana, herb, fern, and cycad. For trees and subshrubs, only the succulent subcategory is given, while for shrubs two subcategories, such as succulent and parasitic, were reported. Similarly, for the herbs ten subcategories were reported: aquatic, climber, epiphyte, geophyte, graminoid, parasitic, rhizomatous, seagrass, saprophyte, and succulent. Available life cycle information (annual, biennial and perennial) was also provided.

For each taxon, occurence locality and geographical distribution within the national border were recorded, based on information provided by the literature sources, on-line databases and herbarium specimen labels. Occurrence localities were organized by province and coded as follows: Maputo City and Province (MP), Gaza (G), Inhambane (I), Manica (MC), Sofala (S), Tete (T), Zambézia (Z), Nampula (NP), Cabo Delgado (CD), and Niassa (NI). If available, data on localities of occurrence, such as protected areas or a specific locality, were also provided. To update the toponyms referring to the colonial period and standardise the Mozambican locality names, a review was conducted through The GeoNames (2021) geographical database (https://www.geonames.org/). Although the geographical information included in the checklist cannot be considered exhaustive, it is provided to support further studies of Mozambican flora.

For endemic and near-endemic species we referred to the criteria in Darbyshire et al. (2019). “Strict-endemic” (E) species were defined as those occurring only within the country borders, while near-endemic (NE) species were designated as those occur in five or fewer localities, besides Mozambique. Endemisms (considering both strict-endemic and near-endemic taxa) were derived from Darbyshire et al. (2019) and through the references databases.

To evaluate the extinction risk of Mozambique vascular plants, The IUCN Red List of Threatened Species (2021) (https://newredlist.iucnredlist.org/) was adopted as the key reference. Taxa were categorised according to the IUCN (2012) categories. An additional remark was included for taxa that need to have Mozambique added to the geographic range in their published IUCN assessments, following the results of this study.

The known vernacular names, obtained from literature sources and herbarium specimen labels, were reported using a simple code composed of the local language and/or the province where it is spoken. The checklist of vernacular plants names compiled by de Koning (1993), has been adopted as a key reference. Although it cannot be considered exhaustive and updated, it represents the most extensive collection of Mozambique’s vernacular plants names. Overall, vernacular names from 18 local languages were reported. All traditional uses of plants recorded in literature sources were included using the following categories: medicine (treatments or remedies for various pathologies), veterinary (animal healthcare), food (preparation of food and beverages, subsistence resources), livestock fodder (plant materials eaten by livestock), cosmetic (personal care), handicraft (production of tools and furniture), fuel (energy supply), ornament (domestic, urban and landscape design), poison (used for hunting purpose) and beliefs (for taxa associated to local beliefs or mystical rituals).

All data were aggregated in a Microsoft Excel 2010 spreadsheet and managed through R software version 3.6.1 (R Core Team 2019).

## ﻿Results and discussion

**Figure 3.**
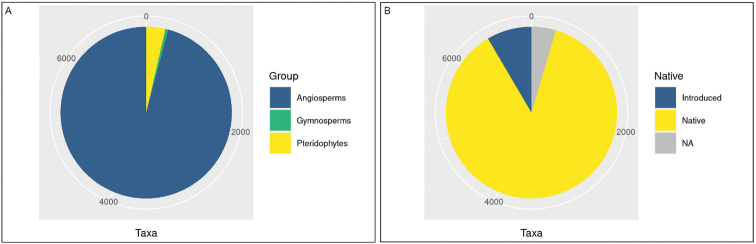
Floristic patterns for Mozambique’s vascular plants. **A** frequency of plant groups **B** geographic origin of taxa.

The updated checklist of Mozambique vascular plants, presented in Suppl. material 1, accounts for 7,099 taxa (5,957 species, 605 subspecies, 537 varieties), belonging to 226 families and 1,746 genera. These findings, significantly increasing the record of 3,932 vascular plant taxa in Mozambique registered in the previous checklist (da Silva et al. 2004), can be related to the recent increase of botanical exploration in the country (Cheek et al. 2018) and to the availability of freely accessible online botanical databases. The larger group of plants recorded is the angiosperms, 200 families and 1,655 genera, representing 95.8% (6,804) of the listed taxa. Pteridophytes, 20 families and 77 genera, amount to 3.6% (257) of the taxa. Gymnosperms, with 6 families and 14 genera, account for only a very small percentage (0.5%; 38) of the taxa (Fig. 3A).

A total of 6,171 taxa (86.9%) are native to Mozambique, while 602 (8.5%) are introduced, mostly for commercial purposes (Syliver et al. 2020). The remaining 326 taxa (4.6%) are assessed as uncertain status (Fig. 3B).

**Figure 4.**
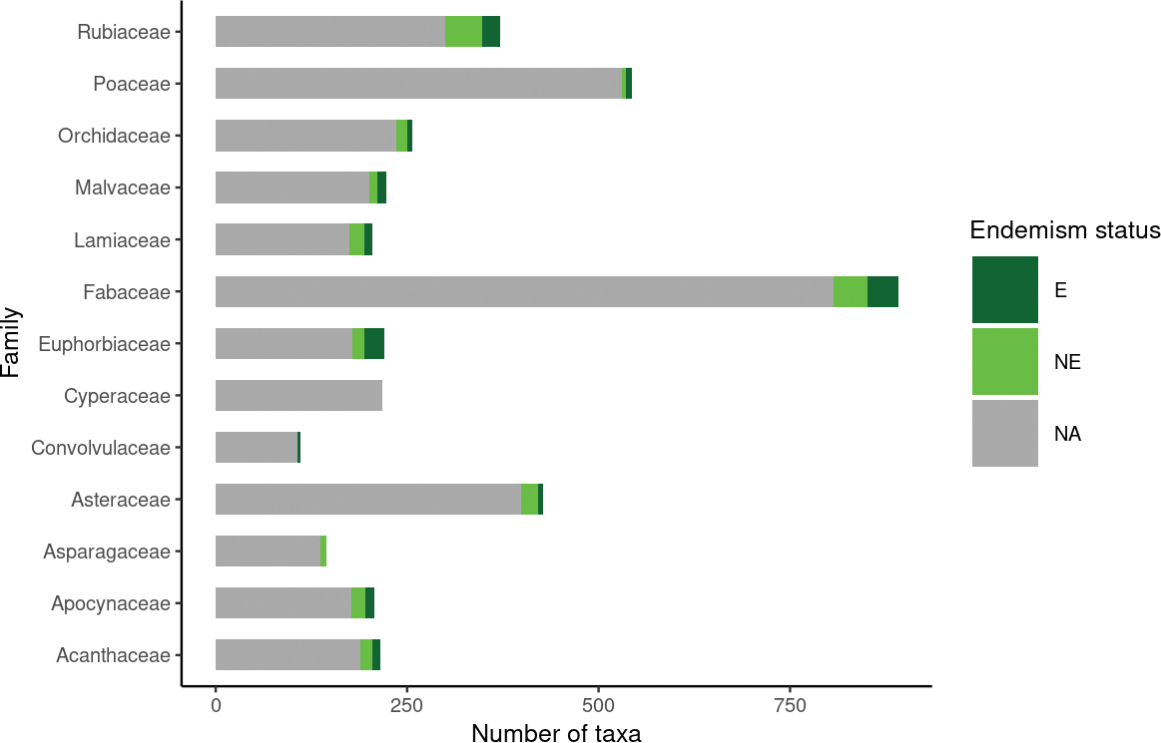
Most represented families and their endemism rate.

As seen in other African countries (Braun et al. 2004; Mapaura and Timberlake 2004; Zhou et al. 2017), the three most taxa-rich families in Mozambique are Fabaceae (891 taxa), Poaceae (543) and Asteraceae (428), which also represent the largest families in the world (Zhou et al. 2017). Other well represented families (≥100 taxa) are: Rubiaceae (371), Orchidaceae (257), Malvaceae (223), Euphorbiaceae (220), Cyperaceae (218), Acanthaceae (215), Apocynaceae (207), Lamiaceae (205), Asparagaceae (145) and Convolvulaceae (111) (Fig. 4). The most taxa-rich genera (≥ 100 taxa) are *Cyperus* L. (Cyperaceae), *Crotalaria* L. (Fabaceae) and *Indigofera* L. (Fabaceae) accounting for 109, 108 and 102 taxa, respectively.

**Figure 5.**
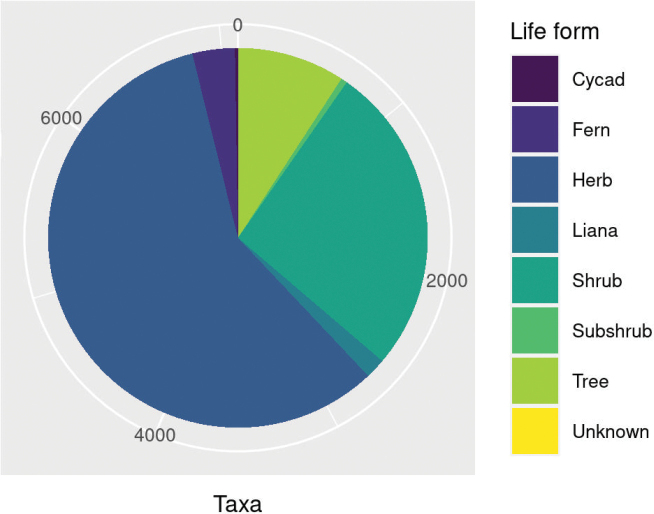
Life form categories.

The majority of taxa in the checklist (58.2%) are herbaceous, while shrubs and trees account respectively for 26.5% and 9.2% of the listed taxa. The other life form amount to 6.1% of the listed taxa, divided into: ferns (3.6%), lianas (1.7%), subshrubs (0.5%) and cycads (0.3%). Due to the lack of reliable data, only 1 taxon was not assigned to any life forms category (Fig. 5).

Table 1 shows all life form categories and the growth habit subcategories and life cycle of trees, shrubs, subshrubs, and herbaceous taxa. 2.5% of tree taxa are classified as succulent. Among subshrub taxa just a small number of taxa are classified as succulent (7.9%), while the shrubs include two growth form subcategories: parasitic (3.5%) and succulent (3.0%). Much growth form diversity is found among the herbaceous taxa, with 10 different subcategories (Table 1). As to life cycle, 66.1% of the herbaceous taxa are identified as perennial, 27.5% as annual and only a small percentage (0.1%) as biennial.

**Table 1.** Life form, growth form and life cycle of the taxa.

Life Form	Growth form	Life cycle	Number of taxa
**Tree**	–	–	**651**
	Succulent	–	16
**Shrub**	–	–	**1883**
	Succulent	–	57
	Parasitic	–	65
**Subshrub**	–	–	**38**
	Succulent	–	3
**Herb**	–	–	**4240**
	–	Annual	1129
	–	Biennial	4
	–	Perennial	2166
	Aquatic	–	54
	Climber	–	276
	–	Annual	3
	–	Perennial	188
	Epiphyte	–	93
	Geophyte	–	148
	Graminoid	–	84
	–	Annual	25
	–	Perennial	59
	Parasitic	–	14
	–	Annual	4
	–	Perennial	10
	Rhizomatous	–	10
	Seagrass	–	9
	Succulent	–	127
	–	Annual	3
	–	Perennial	124
	Saprophyte	–	5
	Unknown	–	10
**Liana**	–	–	**122**
**Fern**	–	–	**257**
**Cycad**	–	–	**18**
**Unknown**	–	–	**1**

Regarding endemic and near-endemic plant species, Mozambique has a total of 278 strict-endemic taxa and 403 near-endemics, giving a total endemism level of 9.6%. The increase in the number of strict-endemic and near-endemic taxa compared previous studies (Darbyshire et al. 2019) can be related to the continuous progress in the knowledge of the Mozambique’s flora. About 56.1% of the listed strict-endemic taxa are included in ten angiosperm families: Fabaceae (40), Euphorbiaceae (26), Rubiaceae (23), Apocynaceae (12), Malvaceae (12), Lamiaceae (11), Acanthaceae (10), Asphodelaceae (8), Asteraceae (7), Orchidaceae (7). Except for Asphodelaceae, these families are also the most represented families in the vascular flora of Mozambique, showing a congruence between the most species-rich families and those with the highest rate of endemism (Fig. 4). The geographical distribution of endemic taxa in this checklist closely matches the findings of Darbyshire et al. (2019), confirming the importance of Mozambique’s Centres of Endemism for flora conservation efforts. Similarly, the mountain areas of the country play a crucial role in the conservation of endemic flora, with the Chimanimani Mountains and Mount Namuli representing the most frequently recorded localities for strict and near endemic taxa, respectively with 137 and 59 taxa. Overall, about 40.2% of endemic taxa (both strict and near endemic) occur in the mountainous areas of the country, consistent with the global pattern of high rates of endemism at high altitudes (Steinbauer et al. 2016).

**Figure 6.**
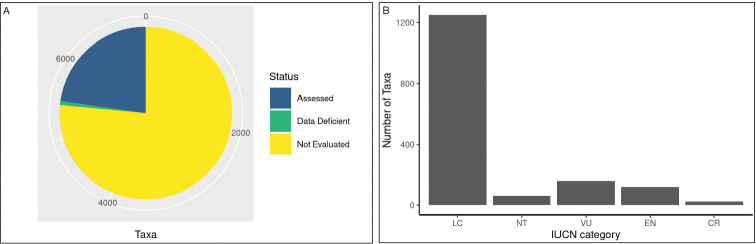
Extinction risk of Mozambique’s vascular plants. **A** assessed taxa **B** IUCN category for the evaluated taxa.

At the time of compiling this checklist (July 2021), 1,667 of the recorded taxa were registered on the IUCN Red List. Overall, the global extinction risk status for 76.5% of Mozambique’s vascular flora is not evaluated (including the taxa categorised as Not Evaluated and those not listed in the IUCN Red List), while a further 0.8% of the taxa are categorised as Data Deficient (Fig. 6A). Such findings highlight the general lack of information on the conservation status of Mozambique’s vascular plants. Further studies are urgently needed to identify threatened species and develop proper conservation strategies. About 18.1% of the evaluated taxa are categorized as threatened: 158 Vulnerable, 119 Endangered, and 24 Critically Endangered (Fig. 6B).

**Figure 7.**
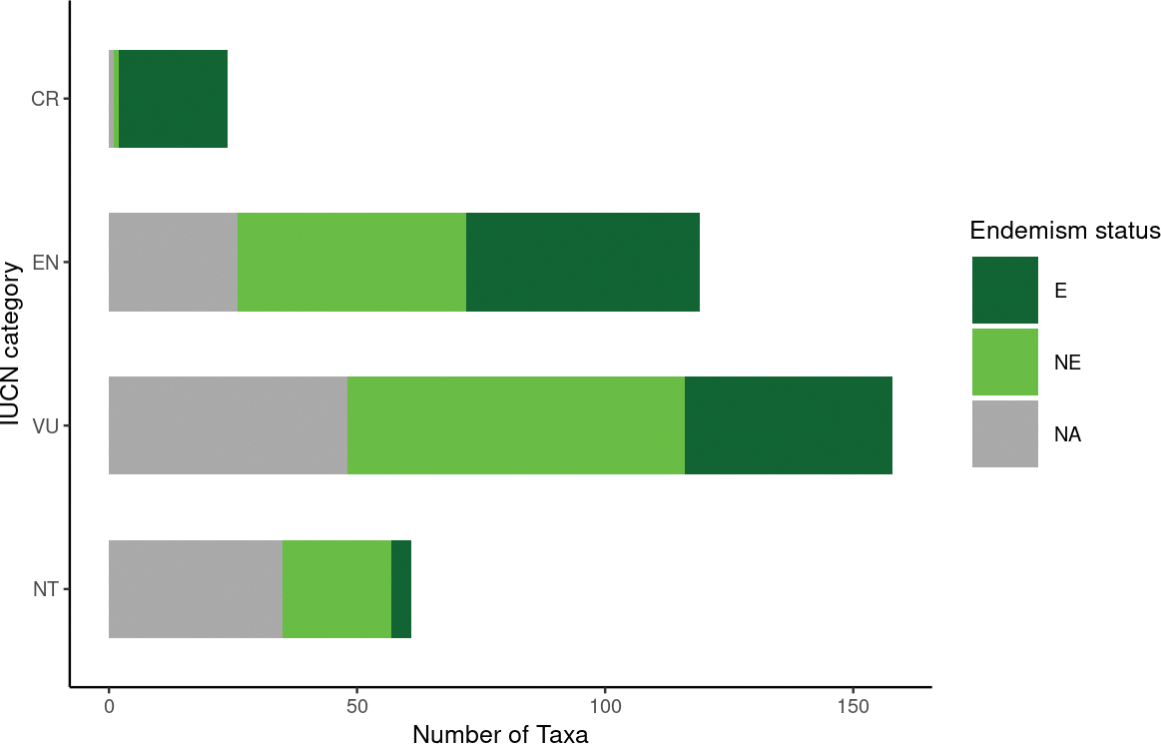
Endemism rate among the threatened taxa.

From these data clearly emerge the need to implement effective conservation strategies for Mozambique’s flora. According to Darbyshire et al. (2019) the main threat factors for vascular plants in the country are habitat loss and degradation, driven by the recent population growth and the consequent increased pressure on natural ecosystems. The growing commercialisation and the over-exploitation of medicinal plants are also becoming an increasing threat (Bandeira et al. 1996; Senkoro et al. 2020). For example, *Warburgiasalutaris* (G. Bertol.) Chiov., one of the most widely used medicinal plants in southern Africa, as a consequence of the increasing commercial demand in the last few years has been subjected to uncontrolled harvesting, resulting in a widespread tree mortality and even in the extinction of local populations in many areas, changing its conservation status to globally Endangered (Senkoro et al. 2020). Moreover, about 75.1% of Mozambique’s threatened taxa (Vulnerable, Endangered and Critically Endangered) are endemic to Mozambique (111 strict-endemic; 115 near-endemic) (Fig. 7), highlighting the central responsibility of the country for the conservation of these taxa.

Finally, we have identified 261 of Mozambique’s vascular plants assessed in the IUCN Red List of Threatened Species, for which Mozambique is not indicated in the geographical range of distribution. Therefore, there is a need to update these assessments, which provide an invaluable tool for plant conservation.

**Figure 8.**
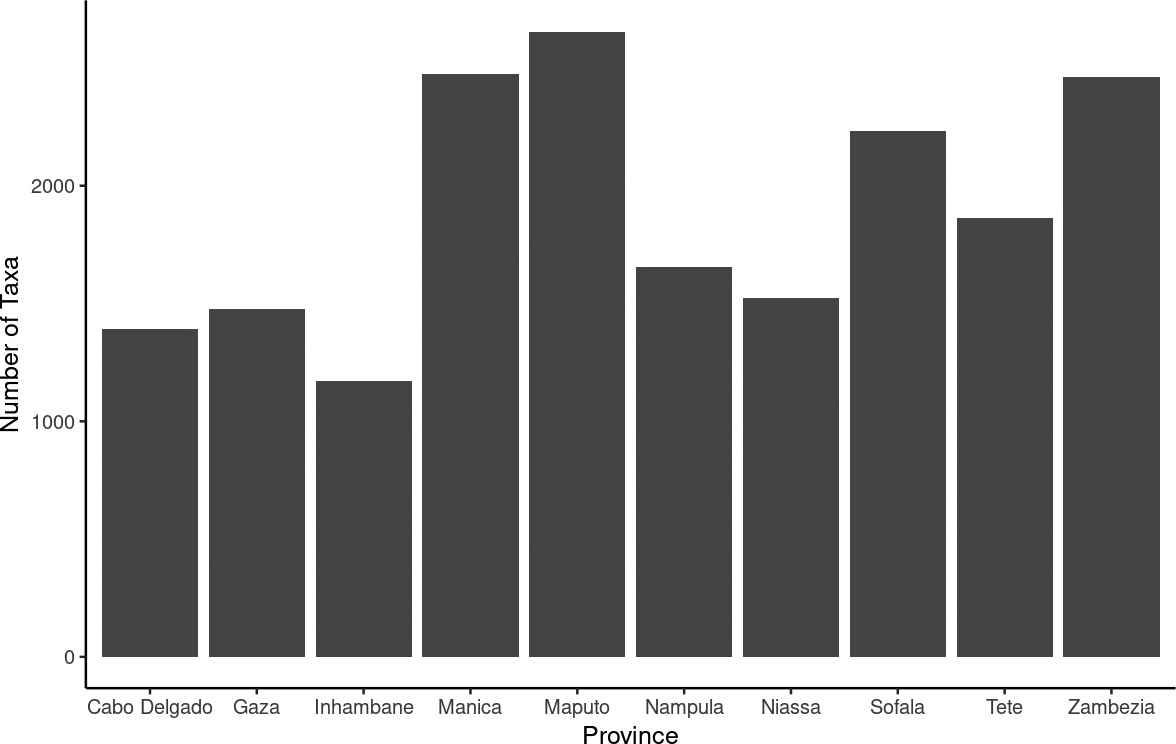
National distribution of occurrence records.

Based on available occurrences, the geographic distribution of Mozambique’s vascular plants is provided in Fig. 8. The distribution patterns identified do not indicate specific latitudinal or regional gradients. Instead, a total of twenty-four occurrence localities, scattered among the province of the country, are recorded. The main occurrence localities (≥ 500 taxa) identified are: the Gorongosa National Park (Sofala province, 740 taxa), the Chimanimani Mountains (Manica, 668), the Serra Gorongosa plateau (Sofala, 549), the Mount Namuli (Zambézia, 536) and the Inhaca Island (Maputo, 534). Other relevant key occurrence localities (ranging from 200 to 400 taxa) are Palma (Cabo Delgado, 316 taxa), Vilanculos (Inhambane, 315 taxa), Serra Chiperone (Zambézia, 298 taxa), Tsetsera & Serra Zuira (Manica, 283), Quiterajo (Cabo Delgado, 255), Mount Mabu (Zambézia, 249), and Serra Choa (Manica, 227 taxa). All occurrence localities are provided in the Suppl. material 1. These localities must be considered of high botanical value for floristic study in the country, making their preservation a strategic priority. A large number of taxa are found in the central provinces of the country (Sofala, Manica, Tete, and Zambézia), which host a total of 4,765 taxa. In the South (Maputo, Gaza and Inhambane provinces) 3,292 taxa are recorded, while the North (Nampula, Cabo Delgado and Niassa provinces) counts 3,120 taxa. The most taxa-rich provinces (≥ 2,000 taxa) are Maputo, Manica, Zambézia, and Sofala, accounting for 2,654, 2,474, 2,461 and 2,231 taxa, respectively (Fig. 8). Although the data collected partially fills the previous knowledge gap on the floristic biodiversity of northern Mozambique (da Silva et al. 2004), the southern and central regions of the country still remain the most widely explored. To properly assess the distribution of plant species in Mozambique, further studies conducted equally throughout the country should be undertaken.

The vernacular names of 1,339 vascular plants of Mozambique are reported in this checklist. Considering the various language spoken in the country (According to de Koning (1993), at least eighteen main local languages excluding Portuguese) such ethnobotanical knowledge can play a pivotal role for research on Mozambique’s plant biodiversity. Indeed, knowing the local names of plants can facilitate investigations carried out in the field with the support of local indigenous people who lack a formal taxonomic knowledge (de Koning 1993).

**Figure 9.**
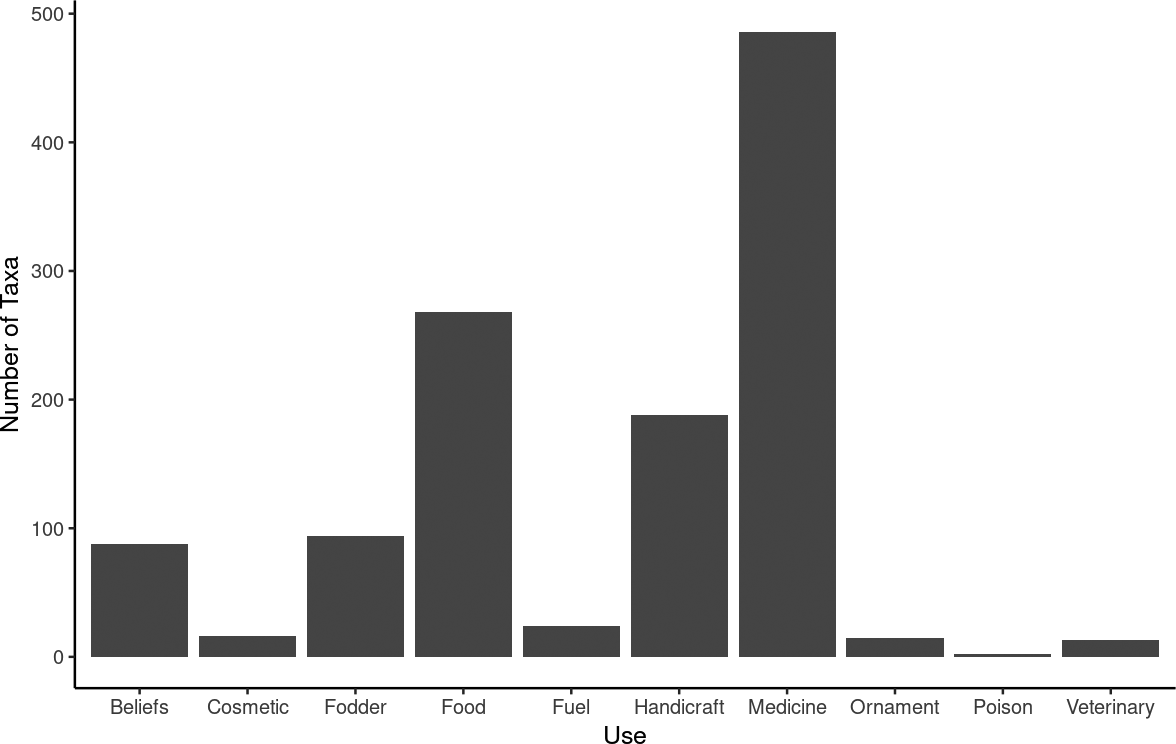
Mozambican vascular plants related to traditional uses.

The review of available ethnobotanical data for Mozambique’s vascular plants resulted in the identification of 773 taxa with traditional uses (Fig. 9), showing the fundamental role played by plants in the livelihood of the Mozambican population. About Sixty percent (62.9%) of these plants are used for medicinal purposes. These account for about 6.9% of Mozambique’s flora, in line with previous estimates (Bruschi et al. 2011) which highlights the importance of traditional medicine in the Mozambican population’s health care. Other significant traditional uses found are as food supply (34.7%), handicraft production (24.3%), and livestock fodder (12.2%). A smaller number of plants are used as ornamental elements (1.9%), fuel (3.1%), cosmetics (2.1%), in the veterinary field (1.7%), and as poison (0.3%). Finally, 11.4% of traditional plants use is associated to local beliefs. A total of 282 plants (4.0% of the listed taxa) are associated with more than one use, such as *Asparagusafricanus* Lam., *Bosciaalbitrunca* (Burch.) Gilg & Gilg-Ben., *Elaeodendronschlechterianum* (Loes.) Loes., *Eucleadivinorum* Hiern., and *Trichiliaemetica* Vahl., all having six recorded use categories. Such a wealth of multipurpose taxa highlights the richness and variety of traditional knowledge related to the use of plants in Mozambique, particularly with regard to traditional medicine (Ribeiro et al. 2010).

## ﻿Conclusion

This is a comprehensive and up to date checklist of Mozambique’s vascular plants, summarizing data from relevant literature sources, herbarium collections and authoritative botanical databases. The reported data, including taxonomic classification, biological and morphological attributes, geographical distribution, endemism, extinction risk, and ethnobotanical information, can represent a reliable basis for further botanical studies in Mozambique. In this respect, future efforts should be focused on maintaining the country’s floristic knowledge so that it is regularly and frequently updated and easily accessible, in order to support botanical research and plant biodiversity conservation in Mozambique.

## Supplementary materials

Supplementary material 1 The updated checklist of Mozambique's vascular plants Data type species data Explanation note The updated checklist of Mozambique’s vascular plants includes 7,099 taxa (5,957 species, 605 subspecies, 537 varieties), belonging to 226 families and 1,746 genera. For each entry in the checklist, the following available data are reported: nomenclature, taxonomic classification, distribution, local occurrence details, life form and life cycle, endemism, extinction risk, vernacular names and traditional uses. https://binary.pensoft.net/file/639339This dataset is made available under the Open Database License (http://opendatacommons.org/licenses/odbl/1.0/). The Open Database License (ODbL) is a license agreement intended to allow users to freely share, modify, and use this Dataset while maintaining this same freedom for others, provided that the original source and author(s) are credited.Délcio Odorico, Enrico Nicosia, Castigo Datizua, Clayton Langa, Raquel Raiva, Joelma Souane, Sofia Nhalungo, Aurélio Banze, Belkiss Caetano, Vânia Nhauando, Hélio Ragú, Mário Machunguene Jr., Jónata Caminho, Leonel Mutemba, Efigénio Matusse, Jo Osborne, Bart Wursten, John Burrows, Silvio Cianciullo, Luca Malatesta, Fabio Attorre
